# Efficacy and safety of acupuncture for the treatment of non-specific acute low back pain: a randomised controlled multicentre trial protocol [ISRCTN65814467]

**DOI:** 10.1186/1472-6882-6-14

**Published:** 2006-04-21

**Authors:** Jorge Vas, Emilio Perea-Milla, Camila Mendez, Luis Carlos Silva, Antonia Herrera Galante, Jose Manuel Aranda Regules, Dulce M Martinez Barquin, Inmaculada Aguilar, Vicente Faus

**Affiliations:** 1 Unidad de Tratamiento del Dolor, Centro de Salud Dos Hermanas "A", Segovia s/n, 41700 Dos Hermanas, Spain; 2 Unidad de Apoyo a la Investigación (Red IRYSS), Hospital Costa del Sol, Ctra Nacional 340, km 187, 29600 Marbella, Spain; 3 Servicio Protección Civil, Delegación de Gobierno, Plaza de España 19, 11006 Cádiz, Spain; 4 Departamento de Investigaciones, INFOMED, Calle 27 e/M y N n°110, Vedado, 10400 Ciudad de la Habana, Cuba; 5 Servicio de Rehabilitación, Complejo Hospitalario Carlos Haya, Avda Dr Galves Ginachero s/n, 29009 Málaga, Spain; 6 Centro de Salud San Andrés-Torcal, José Palanca s/n, 29300 Málaga, Spain; 7 Unidad de Tratamiento del Dolor. Distrito Sanitario, Sevilla 23, 29009 Málaga, Spain; 8 Servicio de Farmacia, Hospital Costa del Sol, Ctra Nacional 340, km 187, 29600 Marbella, Spain

## Abstract

**Background:**

Low back pain and its associated incapacitating effects constitute an important healthcare and socioeconomic problem, as well as being one of the main causes of disability among adults of working age. The prevalence of non-specific low back pain is very high among the general population, and 60–70% of adults are believed to have suffered this problem at some time. Nevertheless, few randomised clinical trials have been made of the efficacy and efficiency of acupuncture with respect to acute low back pain. The present study is intended to assess the efficacy of acupuncture for acute low back pain in terms of the improvement reported on the Roland Morris Questionnaire (RMQ) on low back pain incapacity, to estimate the specific and non-specific effects produced by the technique, and to carry out a cost-effectiveness analysis.

**Methods/Design:**

Randomised four-branch controlled multicentre prospective study made to compare semi-standardised real acupuncture, sham acupuncture (acupuncture at non-specific points), placebo acupuncture and conventional treatment. The patients are blinded to the real, sham and placebo acupuncture treatments. Patients in the sample present symptoms of non specific acute low back pain, with a case history of 2 weeks or less, and will be selected from working-age patients, whether in paid employment or not, referred by General Practitioners from Primary Healthcare Clinics to the four clinics participating in this study.

In order to assess the primary and secondary result measures, the patients will be requested to fill in a questionnaire before the randomisation and again at 3, 12 and 48 weeks after starting the treatment. The primary result measure will be the clinical relevant improvement (CRI) at 3 weeks after randomisation. We define CRI as a reduction of 35% or more in the RMQ results.

**Discussion:**

This study is intended to obtain further evidence on the effectiveness of acupuncture on acute low back pain and to isolate the specific and non-specific effects of the treatment.

## Background

Low back pain and its associated incapacitating effects constitute an important healthcare and socioeconomic problem, as well as being one of the main causes of disability among adults of working age [1]. The prevalence of non-specific low back pain is very high among the general population, and 60–70% of adults are believed to have suffered this problem at some time [2]. Acute low back pain is of non-specific musculo-skeletal origin in 95% of cases [3] and the process is normally self-limiting. However, between 2% and 24% of cases last over three months[4,5]. In Spain, absence from work because of back pain lasts an average of almost 22 days and costs, on average, 1260 euros per worker, representing 19–25% of the total expense of temporary incapacity benefit paid. An average of 55,388 work days are lost every year because of back pain. By sectors, the highest number of days lost for this reason correspond to industry (37.1%), the service sector (29.9%), construction (27.5%) and agriculture (5.5%). About 90% of patients affected by non-specific back pain resume work within 6 months [6].

Apart from the standard distinction between the acute nature of low back pain (less than 6 weeks' duration) [1], subacute (between 6 weeks and 3 months) and chronic (more than 3 months), the various texts on the question have stressed the need to distinguish between specific and non-specific low back pain. There is generalised agreement that the clinical history and the physical examination should be sufficiently detailed to enable a diagnostic orientation and to flag the existence, if any, of the so-called red flags, such as fractures, tumors, infections or inflammatory rheumatic illnesses such as ankylosing spondylitis, as well as factors that may promote the chronic aspect of the process, the so-called yellow flags, or factors that are individual and work-related [3].

The conventional standard treatment (CT) for non-specific acute low back pain is to prescribe medicaments such as non-opiate analgesics, non-steroidal anti-inflammatory drugs (NSAIDs), myorelaxants and opioides. The evidence obtained from the 51 studies included in a systematic review [7] suggests that NSAIDs are effective for the short term relief of symptoms among patients with acute low back pain, but that there does not seem to be any one specific type of NSAID that is clearly more effective than any other. Myorelaxants are effective in treating non-specific low back pain, but their adverse side effects mean they must be used with caution. What is needed are trials to determine whether myorelaxants are more effective than analgesics or NSAIDs [8]. It has yet to proven whether benefit is to be derived from physical fitness programmes in reducing work time lost and in increasing the functional state of patients who suffer from acute low back pain [9].

Few randomised clinical trials (RCT) have been made to assess the efficacy of acupuncture in treating acute low back pain. A recent review by Cochrane Collaboration selected just three studies for analysis, and was unable to draw conclusive conclusions because of the small size of the samples and the poor methodological characteristics of the studies examined [10]. A later review [11] that extended the search area to August 2004 was no more conclusive as regards acute low back pain.

The different RCT that have been made to investigate the efficacy of acupuncture tend to use different types of control group, with the following being the most commonly adopted: 1) sham acupuncture, which has various possible modes, including: A) performing acupuncture at points that are not considered to be acupuncture points, to a depth of less than 2 mm and without applying any stimulus to these points; B) performing acupuncture at points that are considered to be acupuncture points but which are not specifically recommended for treating the pathology in question; 2) placebo acupuncture, which consists of using retractable telescopic needles resting on an adhesive base that exercises a pressure on the skin without actually puncturing it; 3) alternatively, in situations in which the patient cannot observe the technique directly (e.g. points on the patient's back, with the patient lying face down), what is known is acupuncture simulation, which consists of pressing with a blunt object (or a pointed one such as a toothpick), on the surface of the skin, making the patient believe that a needle has been inserted.

Complex non-pharmacological treatments, such as acupuncture, are complicated to evaluate because it is difficult to isolate the characteristic or specific effects of the technique from the non-specific ones (i.e. the placebo effects) [12]. One problem that arises in investigating the problem is that of choosing appropriate placebo controls if the goal is to evaluate specific effects [13]. For example, techniques that involve the puncture/penetration of the skin and which present the lowest probability of being discovered, and thus are considered the best in terms of the degree of blinding achieved, may cause a significant physiological response [14]. Various reviews have described a negative correlation between the quality of the study and the results obtained (i.e. the highest quality studies tend to produce negative results more often [15-17]. This finding should be interpreted with caution because many revisions include trials with non-comparable control groups, such as a waiting list, or a group that received no treatment at all, or different types of placebo (such as those listed above) and a variety of active control groups.

Studies in which the results are compared for different control groups with different types of placebo may make things clearer, in separating non-specific or contextual effects from those derived specifically from the application of acupuncture.

In the light of these considerations, we designed a randomised controlled study of patients with non-specific acute low back pain in order to investigate the efficacy of traditional acupuncture in comparison with three types of control group (sham acupuncture, placebo acupuncture and conventional treatment) with the objectives of isolating the effects attributable to each of these treatments and of separating the specific from the non-specific ones. We also sought to assess the evolution of the illness, using a system to measure the patient's degree of incapacity, the intensity of the pain suffered, the quality of life, the duration of the absence from work, the consumption of analgesic and anti-inflammatory medication and the potential effect of the factors that were considered to be predictors of a negative outcome [18], and to carry out a cost-effectiveness analysis.

## Methods/Design

### Design

Randomised four-branch controlled multicentre prospective study made to compare semi-standardised real acupuncture (Group A), sham acupuncture (acupuncture at non-specific points) (Group B), placebo acupuncture (Group C) and solely the conventional treatment recommended in guides to clinical practice [19,20] (Group D). The patients are blinded to the real, false and placebo acupuncture treatments. The evaluation of the patients and the analysis of the results will be carried out by professionals who are blinded to the assignment of patients to the various groups.

### Study subjects

Persons of working age who attend for treatment, for the first time in at least six months, reporting symptoms of non-specific acute low back pain to their General Practitioner (GP) at one of the Primary Healthcare Clinics participating in the study and belonging to the Andalusian Public Health System (Pain Treatment Unit at the Dos Hermanas Healthcare Clinic in Sevilla, Rehabilitation Service at the Specialised High Resolution Centre in Malaga, the Acupuncture Unit of the Málaga Health District and the San Andrés-Torcal Healthcare Clinic in Málaga). The patients will be informed as follows: "This Centre is engaged in a study intended to evaluate and quantify the effects of conventional treatment, in isolation, compared to the same treatment applied in combination with two types of acupuncture, one of these being similar to traditional Chinese acupuncture, and the other not following the same principles. In addition, there will be a control group which will be given placebo acupuncture (in addition to the conventional treatment). I have been told that I may be included in a random fashion (by means of a draw) in one of the four treatment groups". The patients will also be informed of the possible risks associated with the different types of acupuncture (infection, fainting, bruising) and that they may end their participation in the study at any time without suffering any kind of penalisation or loss of benefits to which they would otherwise be entitled.

### Selection criteria

▪ Criteria for **inclusion**:

• Signature of informed consent form;

• New episode of non-specific acute low back pain of less than 2 weeks' evolution, with or without irradiation (diagnosed by clinical history and physical examination). We define 'new' as the first episode in at least the last 6 months.

• Patient of working age (whether in paid employment or not), either occupationally active or absent from work because of back pain;

• No previous treatment with acupuncture (in order to minimise the possibility of patients being able to distinguish the real acupuncture treatment from the various control modes).

▪ Criteria for **exclusion**, one or more of the following symptoms or illnesses:

• More than one absence from work because of back pain within a period of 6 months (in order to eliminate possible mercenary motives);

• The presence of alarm signs that suggest the protrusion or prolapse of one or more intervertebral disks with concurrent neurologic symptoms, infectious spondylopathy, previous surgery affecting the spine, low back pain caused by inflammatory illness, whether malign or autoimmune, congenital deformities of the spine except for slight scholiosis or lordosis, vertebral fractures, stenosis of the spinal canal, spondylolysis or spondylolisthesis ;

• Contraindications for acupuncture such as extensive skin disorders, treatment with anticoagulants, or pregnancy;

• Incapacity to complete the questionnaires or to answer the questions of the assessor.

### Ethical criteria

The ethical validity of this study has been analysed by the Andalusian Regional Committee for Clinical Trials, after approval by the Research Committee at each of the healthcare clinics concerned. In designing this study, due attention was paid to the fundamental principles established in the Helsinki Declaration, as restated in Tokyo 2004, at the Convention of the Council of Europe concerning human rights and biomedicine, as well as the requirements set out under Spanish law with respect to biomedical research, the protection of personal data and bioethics. All the patients must sign a statement of informed consent to the clinical procedures involved in the study. During its development, we shall carry out the audits required by the Research and Ethics Reference Committee at each clinic, in addition to any external audits (of the research fund provider) that may be required.

### Randomisation

Sampling will be by consecutive selection according to criteria of inclusion-exclusion during a period of 12 months until the required sample numbers are achieved. The randomised allocation to the four branches of the study will be performed using a specialised computer program for this purpose (EpiDat v 3.0) at a central location (University of Sevilla, School of Psychology, Department of Social Psychology), following a 1:1:1:1 pattern (true acupuncture: sham acupuncture: placebo acupuncture: conventional treatment), with one sequence for each clinic, in blocks of eight. Neither the clinics nor the doctors participating in the study will be involved in the randomisation process. The patients who meet the criteria for inclusion and who give their written informed consent will be included in the study. Following inclusion, the patient's GP will contact the randomisation centre, where the patient will be registered.

### Participating clinics and doctors

The patients will be referred to one of the four reference clinics for the baseline assessment to be performed, after confirmation, on the referral form provided, that the criteria for inclusion are met and that conventional treatment has been prescribed by the corresponding GP. The four clinics within the Andalusian Public Health System that are included in the study are:

1. Pain Treatment Unit at the Dos Hermanas "A" Health Centre (Sevilla)

2. Rehabilitation Service at the Specialised High Resolution Clinic (Málaga)

3. Acupuncture Unit of the Málaga Healthcare District (Málaga)

4. San Andrés-Torcal Health Centre (Málaga)

**Figure 1:**
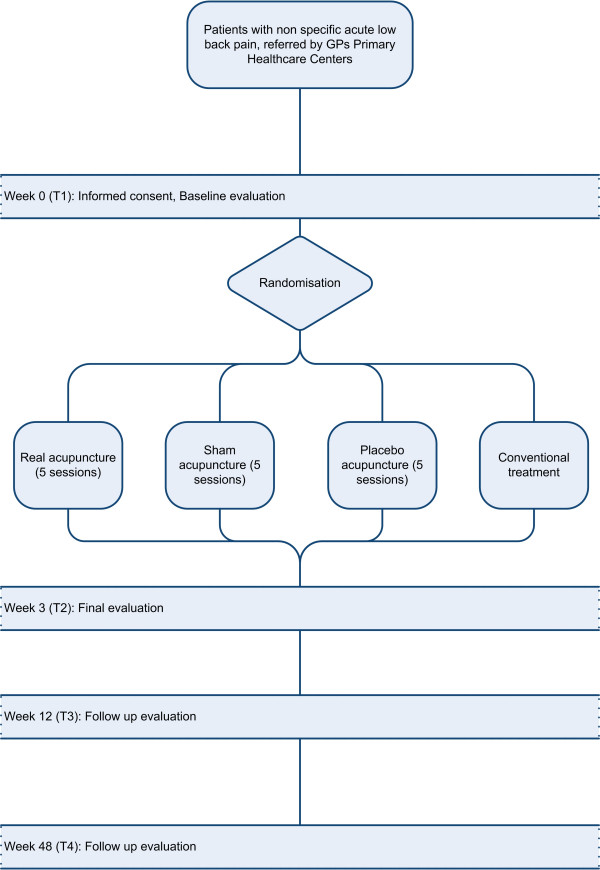
Study sequence

At these clinics, the baseline assessment will be carried out by independent assessors who will explain the procedure and invite the patients to give their informed written consent and to fill in a set of self-report questionnaires. Following this, they will enter the treatment room where the doctors responsible for the treatment will make an assessment according to the principles of traditional Chinese medicine (TCM). They will then contact the Randomisation Centre to receive the patient's assignment to one of the study groups and, in the case of those belonging to Group B, the randomised sequence of points. Those patients assigned to Groups A, B and C will be treated with the corresponding acupuncture technique (true, sham or placebo), always by the same doctor. The true, sham and placebo acupuncture treatments will be blinded to the patient and the assessor, but this cannot be done for the doctor who must apply the treatment. To maximise the blinding of the participants, we shall only include patients who have no previous experience of acupuncture, so that they will be unable to compare this treatment with earlier ones; moreover, contact between participants in the study will be restricted. At the end of the treatment, data will be compiled to determine the effectiveness of the blinding. The sequence to be followed in the study is set out in Figure 1. The doctors who will perform the different treatments have all received at least 700 hours' training and have an average experience of 8.5 years in the field.

### Treatment details

The true, sham and placebo acupuncture groups will receive a total of five acupuncture sessions (three in the first week and two in the second), using the technique and the acupuncture points described below. The disposable sterile needles are manufactured by Cloud & Dragaon Radical Device Co., Ltd (Wujiang, China) according to EU standards and are imported by Acupuncture-Shop, Storegade 58, 6800 Vade (Denmark). Any adverse reactions or collateral effects will be recorded in the Data Record Book (DRB) with a detailed explanation of their type and the dates on which they occurred.

It is well known that the expectations of the person administering the technique play an important role, whether the treatment is merely a placebo or genuine, and that an enthusiastic, positive and empathetic attitude reinforces the therapeutic benefits [21]. Therefore, the practitioners who participate in this study will be trained in the different procedures to be employed in order to make them more at ease in the application of the three techniques specified for this study, and so that their attitude shall be as identical as possible in all cases. For the same reasons, the treatment time devoted to the patients assigned to each of the groups (A, B and C) must be identical, as is also the case for the pre- and post-session assessments. We will take the precaution of asking the patients to attend at different times, to prevent them from exchanging opinions and telling each other of their experience. The fulfilment of the protocol will be carefully controlled throughout the duration of the study.

### A) True acupuncture (TA)

Conventional treatment (CT) + semi-standardised acupuncture according to the characteristics of the pain. When the principles of TCM are observed, acupuncture is an individualised therapy. In this study, we designed a flexible, semi-standardised treatment protocol, similar to that used in other studies [22,23], by selecting a group of basic points (BP) that are predefined to be applied in every case, and other, specific points (SP) determined according to the main characteristics of the low back pain suffered by the patient, but allowing the participating doctors the freedom to add individualised points (IP) as advised by their clinical experience or in the light of an individual syndrome, according to the diagnostic principles of TCM. The predefined BP have been carefully selected by a process of consensus with experts from the two Spanish acupuncture societies, taking into account the principles of TCM. The main reason for choosing a semi-standardised treatment is to promote transparency and a certain degree of replicability without sacrificing the aspect of individualisation.

The doctors responsible for applying the treatment will insert a sterile, disposable filiform acupuncture needle of gauge 25 mm × 0.25 mm, or of 40 mm × 0.25 mm, depending on the zone to be punctured, with the aid of a guide tube, at each of the points indicated below, after having disinfected the skin and with the patient lying face down or on his/her side. The puncture will be perpendicular, except in the cases described otherwise, to a depth of 0.5–1 distance (the measurement unit in acupuncture is the *cun*, the equivalent to the width of the patient's thumb) The insertion is followed by stimulation using broad bidirectional rotation movements of the sleeve of the needle in order to produce *Deqi*, which is commonly described as a radiated sensation. The needle is maintained in place for 20 minutes, and rotated for one minute every five minutes (i.e. four times per session). After the BP and the SP have been punctured, *Xingzhen *manipulation (dynamic puncture) will be performed; this consists of asking the patient to flex, extend and rotate the waist, always to the degree that the pain suffered allows, while the above-described manipulation is carried out.

▪ Obligatory BP: N-UE-19 (Yaotongxue), ipsilateral

▪ SP, according to the location of the low back pain:

• Dumai: *Houxi *SI-3, *Shenmai *BL-62 (laterality depending on the type), *Renzhong *GV-26

• Zutaiyang: *Yanglao *SI-6, *Kunlun *BL-60, *Zanzhu *BL-2 ipsilateral

• Zushaoyang and Daimai: *Waiguan *TE-5, *Zulinqi *GB-41, *Yanglingquan *GB-34

▪ IP: a maximum of four points (unilateral and bilateral) following the criteria of the doctor applying the treatment, and on the basis of the diagnosis previously made. These points will be recorded in the DRB.

### B) Sham acupuncture (SA)

CT + acupuncture at points considered non-specific for the complaint being studied [24], selected in a random fashion. This technique has been employed as a control placebo in non pain-related situations. The choice of this method for our study is based on the well known physiological activity of any acupuncture stimulus, namely the production of a certain degree of analgesia by nociceptive inhibition at the rear medullary horn, and even at higher levels, particularly in the brainstem, a phenomenon known as diffuse noxious inhibitory control (DNIC) [25,26]. This technique is associated with a high degree of credibility but, strictly speaking, it should not be considered a placebo. It shares with real acupuncture the non-specific effects such as the time required, the physical contact between patient and doctor, the patient's expectations and the non-specific analgesic phenomena of the puncture. The only difference between this and real acupuncture is that the points to be selected are not ones that are specifically recognised for the treatment of acute low back pain.

The technique employed will not vary in any way from that employed for Group A. When the patient is assigned to Group B, the randomising centre will send a random sequence with four pairs of points that are coded in its database as "non specific" and these will be used in each of the five sessions corresponding to the treatment. These points are as follows:

▪ *Kongzui *LU-6

▪ *Yuji *LU-10

▪ *Zhouliao *LI-12

▪ *Shangqiu *SP-5

▪ *Neiguan *PC-6

▪ *Jianshi *PC-5

▪ *Zuqiaoyin GB-*44

### C) Placebo acupuncture (PA)

CT + placebo acupuncture (pressure in the lumbar region), leading the patient to believe real acupuncture is being applied [27,28]. This technique consists of applying momentary pressure with a semi-blunted needle fitted within a plastic is placed over one of the eight points described below, after disinfection of the skin and with the patient adopting a face-down or lateral position. This technique has been found to be indistinguishable from true acupuncture among patients who suffer acute low back pain and who have never previously been treated with acupuncture [29] and among patients with dental pain [28]. It shares with real acupuncture the non-specific effects of the latter, such as the time employed in the treatment, the physical contact between patient and doctor, and the patient's expectations, but it does not provide the non-specific effects of the puncture that are described for Group B. The patient must remain in a face-down or lateral position for the 20 minutes of the session so that the placebo technique may remain undiscovered. Every five minutes, the doctor responsible for the treatment will repeat the above-described activity over the points in question. For each point, the doctor must open a new, sealed pack containing the sterile needle, to reinforce the credibility of the procedure.

▪ P1: located bilaterally at 1 *cun *from the L1 spinous apophysis

▪ P2: located bilaterally at 1 *cun *from the L2 spinous apophysis

▪ P3: located bilaterally at 1 *cun *from the L3 spinous apophysis

▪ P4: located bilaterally at 1 *cun *from the L4 spinous apophysis

### D) Conventional treatment (CT)

These patients will be assessed in the same way as Groups A, B and C, according to the principles of TCM, after which their inclusion in the conventional treatment group will be explained to them. For all the groups, this treatment will be prescribed and controlled by the corresponding GP. The patients will be asked to return to the clinic after three weeks for a final assessment to be made.

Agreement will be reached with the GPs participating in the study concerning the treatment protocol, as set out in the guidelines to Clinical Practice and Teaching Materials published by the Catalan Health Institute [19] and by the European Group for the Management of Acute Non-specific Low Back Pain in Primary Care (COST B13) [20]. The guidelines will be identical for all the patients entering the study, as follows:

▪ The patient will be informed about his/her situation, seeking to present it as a non-dramatic case.

▪ The patient will be recommended to avoid remaining in bed and to keep as active as possible.

▪ Recommendations will be made concerning the appearance of warning signals.

▪ The agreement reached is that the following drugs may be prescribed:

• Paracetamol (500 - 650 mg/6 - 8 h (maximum 4 gr/day))

• Ibuprofene (400 - 600 mg/8 h)

• Diclophenac (50 mg/8 - 12 h)

• Ciclobenzaprin (10 mg/6 - 8 h (max. 1 week))

Consumption of these drugs will be recorded, together with the pattern of use, in the patient's referral form.

### Variables

#### Selection of variables

To evaluate the primary and secondary result measures, the patients will be asked to fill in a series of questionnaires containing the details described below, before randomisation and also after weeks 3, 12 and 48 after starting the treatment. All the clinical variables will be evaluated and analysed by independent observers. The **primary **result measure is the **clinical relevant improvement **(CRI) of the lumbar complaint at three weeks after randomisation. We define CRI as a reduction of 35% or more in lumbar incapacity as reported on the Roland-Morris Questionnaire (RMQ) [30].

The **secondary **result measures are: CRI at 12 and 48 weeks after randomisation and a series of result measures (pain intensity, improvement perceived by the patient, incapacity to work, quality of life (EuroQol 5D) and consumption of analgesics) used to reflect the multidimensional nature of the impact of low back pain, obtained at 3, 12 and 48 weeks after beginning the treatment. Other **secondary **measures to be used are: a control scale of the credibility of the treatment after the first week of treatment, for Groups A, B and C, the pain intensity before and immediately after each of the treatment sessions, the record of the collateral effects and adverse reactions that may appear up to week 3 (during the treatment phase), the number of new episodes of low back pain reported at weeks 12 and 48, and the number of days' enforced absence from work because of low back pain from the date of final assessment to weeks 12 and 48.

#### Roland-Morris Questionnaire on Lumbar Incapacity (RMQ): primary result variable, clinical relevant improvement (CRI)

The validity of the Spanish version of the RMQ has been confirmed independently [31]. It consists of 24 pages related to the incapacity provoked by low back pain. The patients must mark each question according to whether they consider it may be applicable to their case or their current state (on the day when the questionnaire is filled in). Scoring the questionnaire is fast and straightforward: each question marked is scored one point, and so the accumulated points range from zero (no incapacity caused by low back pain) to 24 (maximum possible incapacity) [32]. This continuous variable will be recoded into a binary variable in order to determine whether CRI has been achieved. The following binary values will be adopted: (0) = Improvement (reduction of 35% or more on the RMQ [30]); (1) = Reduction of less than 35% on the RMQ).

#### Pain intensity, according to a visual analogue scale (painVAS)

There exists ample evidence for the validity of the visual analogue scale (VAS) of pain intensity. Many studies have shown the validity of the construct [33] and its reliability [34,35]. This is a fast and straightforward method of evaluating the subjective intensity of pain. The patient is asked to mark, on a millimetric scale from zero (absence of pain) to 100 (the worst pain imaginable), the degree of intensity of low back pain, both at rest (painVAS-r) and in movement (painVAS-m) experienced on the day the assessment is performed. Data have been obtained showing that reductions of more than 35 mm are associated with patients' sensations of improvement [18].

#### Questionnaire on Fear and avoidance beliefs arising from low back pain (FABQ)

This is the Spanish version of a questionnaire [36] designed to evaluate attitudes of fear and avoidance that are provoked by patients' beliefs concerning the origin and risks of their low back pain. It consists of 16 items divided into two subscales. The first five items assess feelings and attitudes towards physical activity, and the remaining 11 concern the situation with respect to work [37]. The items are measured on a 7-point Likert-type scale, ranging from 0 (total disagreement) to 6 (total agreement), with the final range of possible values extending from zero to 96. The higher the value recorded, the higher the degree of fear and avoidance conduct caused by low back pain. A high degree of reliability has been reported for the FABQ applied to patients suffering acute low back pain [38,39].

#### Improvement perceived by the patient (IPP)

A Likert-type scale of 7 points is recommended for evaluating the improvement perceived by the patient with non-specific low back pain [40], but opinions differ concerning the categories to be used. We have opted for the model proposed by Hudak and Wright [41]:

"How satisfied are you with the results of your recent treatment for low back pain?

1 = Extremely satisfied

2 = Very satisfied

3 = Moderately satisfied

4 = Indifferent (an approximately equal degree of satisfaction and dissatisfaction)

5 = Moderately dissatisfied

6 = Very dissatisfied

7 = Extremely dissatisfied.

#### EuroQol 5-Dimension (EQ-5D)

This is a generic questionnaire, the validity of which has been confirmed in Spain [42], that measures the quality of life as regards personal health. It consists of two parts: in the first, the patient evaluates in a descriptive way his/her health state, with respect to five dimensions, namely mobility, personal care, daily activities, pain/discomfort and anxiety/depression. Each dimension is scored from one to three, and so the best possible health profile is 11111 and the worst is 33333. In the second part, the patient marks on a VAS from zero (the worst imaginable state of health) to 100 (the best imaginable state of health) his/her overall state of health on the day the questionnaire is completed. The two scores are complementary. EQ-5D has an index of reference values of possible health profiles ranging from a value of one (the best state of health) to zero (death). Thus, we seek to combine these results with the years of life in order to calculate the years of life adjusted for health-related quality of life [43-45]. By this approach, as well as analysing cost-effectiveness, we hope to carry out a cost-utility analysis.

#### Consumption of analgesics (CA)

Analgesic medication and NSAIDs consumed (whether or not prescribed by the patient's doctor), scored on a 5-point Likert scale: 0 = none; 1 = less than the normal amount; 2 = daily, at the normal dose; 3 = higher than the normal dose; 4 = in addition to that prescribed, the consumption of other medication. The names and daily doses of the pharmaceutical preparations consumed by the patient will be recorded.

#### Control of the placebo (credibility)

▪ Expectation and Treatment Credibility Scale (ETCS) [46]. This scale was first proposed by Borkovec and Nau [47] and includes four items to be valued on a continuous VAS of 0–10 (0 = totally disagree; 10 = totally agree): (1) I am confident this treatment will alleviate my pain; (2) I consider the treatment a logical one; (3) I would recommend this treatment to a friend or relative suffering the same complaint; (4) I believe this treatment would be an option to consider for treating other problems. This evaluation will be made after the second treatment session, among Groups A, B and C.

▪ Verification of the blinding, with respect to the patient [28]. After the final treatment session for Groups A, B and C, the patients will be asked, "Which treatment do you think you have been given?" The possible answers are: 1 = Real acupuncture; 2 = Sham or placebo acupuncture; 3 = Not sure.

#### Collateral effects and adverse reactions (CEAR)

Possible collateral effects and adverse reactions that may arise as a result of the treatment will be recorded.

#### Sociodemographic variables

The following sociodemographic variables will be recorded: date of birth, sex, marital status (living alone or cohabiting), educational level (no schooling/primary/secondary/university education) and intensity of physical activity at work (high/moderate/sedentary). The level of activity is classified according to risk factors such as the movement or lifting of heavy weights, body posture involving ventral flexing of the spinal column by 45° with the knees unbent, and work with vibratory machinery; if one of these factors is present during more than 50% of the working day, the level of physical activity is considered high; less than 50% is considered moderate and if it is absent, the work is classed as sedentary [6].

#### Variables concerning the severity of the condition and work-related aspects

▪ The duration of absence from work is expressed as days of absence because of the low back pain recognised at the baseline assessment. As a secondary result measure, we shall quantify the duration of transitory incapacity for work caused by low back pain, from the start of the treatment until the 12 and 48-week assessments [18].

▪ Low back pain related to occupational activity (common occurrence or work-related occurrence: Do you think your episode of low back pain is directly related to your work activity) (Yes/No)

▪ The patients will also be asked about their degree of satisfaction with their work, according to a 7-point Likert scale, ranging from zero to six, as follows: 0 = very dissatisfied; 1 = moderately dissatisfied; 2 = a little dissatisfied; 3 = indifferent; 4 = a little satisfied; 6 = very satisfied.

▪ Duration of the present condition of low back pain (in days).

▪ We will also examine whether there is a sensation of irradiated pain; and if so, whether this extends beyond the knee.

▪ The consumption of healthcare resources from the onset of the low back pain (number of times treated by his/her GP, number of times treated at the hospital's emergency department, number of times treated by a specialist, by a private doctor or by a company doctor).

▪ Previous episodes. Patients will be asked, "With respect to the acute low back pain you are presently suffering, is this the first occurrence? (Yes/No). If not, please answer the following:

• How many episodes of low back pain have you had during the last two years?

• How long (in weeks) have you been off work because of your back pain, during the last two years?"

#### Characteristic diagnostic variables, according to traditional Chinese medicine (TCM)

The doctor participating in the study should examine the patient's clinical characteristics in order to make a diagnosis on the basis of which points can be selected for the treatment of acute low back pain. Although these procedure is only of use for the individuals included in Group A, it will be carried out for all the patients involved in the study.

#### Estimate of direct tangible costs

To estimate the costs of the different approaches to treatment (i.e. acupuncture vs. conventional treatment), we shall make a chart of the activities carried out, measure the costs of each and identify areas of difference. The activities will be evaluated according to the Activity Based Costing (ABC) approach, under which each activity consumes resources (inputs), related to personnel, work-absence, material and pharmaceutical costs, in order to produce results (outputs). These activities will be valued on a unitary basis. The cost of each therapeutic intervention is defined as the sum of the costs of each of the activities of which it is constituted. Thus, we will be able to achieve a much more accurate view of the cost of each of the interventions with respect to the other, and can therefore study its impact during the follow-up sub-process and in future studies. The cost components of the activities that are considered and the criteria for their economic evaluation are as follows:

▪ **Personnel**: this covers the consumption of human resources as a result of the development of the different activities. This evaluation includes the cost per hour of each category of professional. The cost will be estimated taking into account the data provided by the Andalusian Public Health System (SSPA).

▪ **Work absence**: We shall quantify the costs arising from temporary occupational incapacity, depending on the type of incident. The number of health-related absences from work from the start of the study until the final follow-up evaluation will be recorded, as will the number of days off work during each such absence.

▪ **Materials**: This includes the consumption of materials during the treatment process. Each material cost will be evaluated at the price stated in the catalogue of the clinic's stores office/Hospital supply division/Health District.

▪ **Pharmaceutical costs**: We will assess the costs of the different pharmaceutical products (number and unit cost), on the basis of the prevailing retail prices.

## Data records and analysis

### Data records

A referral form has been created, to be filled in by the GP requesting the inclusion in the study of patients who meet the selection criteria. We will design a database for the electronic storage of the data recorded in the paper-format Data Record Book (DRB) kept for this purpose. This database will remain at the analysis centre (Research Support Unit, Costa del Sol Hospital, Marbella, Málaga), which will be independent of the randomisation centre. Each participating clinic will have a replica of the structure of this database (DRB-Rep) and a codebook with the definitions and operational characteristics of all the variables. The information will be recorded in a general questionnaire that covers each and every one of the variables considered in the study, both for the self-administered formats and in those obtained by direct observation. These data will be recorded on a daily basis in the DRB-Rep and sent (encoded) to the analysis centre once weekly, to ensure their safety. Neither the DRB nor the DRB-Rep will state which group a patient has been assigned to. The assignment to the different study groups will be known to the doctors who perform the corresponding treatments, and who is responsible for communication with the randomisation centre. Every two weeks, the analysis centre will carry out a quality control exercise on the data received.

### Statistical analysis

The analysis will be carried out for two types of population: (1) intention to treat (ITT) for all the randomised patients; (2) per protocol (PP), including only those patients with minor deviations from the protocol. All the demographic, clinical and baseline result-variable data will be analysed descriptively, using means and standard deviations for the continuous variables and percentages for the categoric ones, performed separately for each treatment group.

The verification of the principle result variable and all the principle analyses will be based on the ITT population. For the principal objective, the null hypothesis to be verified will be that real acupuncture = conventional treatment.

The two-tailed level of significance used will be α<0.05. To adjust for possible confounders and to detect potential interactions with the "treatment group" variable, logistic regression models will be constructed, adjusted for the baseline level. These models will include the variables of the group and the baseline variables, as well as sociodemographic data (age and sex) and of the baseline severity of the complaint (RMQ, painVAS, EQ-5D, CA, FABQ, level of physical activity, duration of time off work), using criteria of statistical significance and of confusion (assuming a change of greater than 10% in the Odds Ratio of the "treatment group" variable). The model will be reconstructed removing the observations with Cook distances greater than the 90 percentile of the distribution, in order to test the consistency of the results. A Kaplan-Meier survival analysis will be performed to evaluate the number of work days lost from the start of the study until the return to normal work activity, using the corresponding Cox's regression model of proportional risks to control for confounders.

Finally, a Bayesian analysis will be carried out on the CRI, principal result variable. This latter analysis is aimed at complementing the conventional analysis with a new approach that could provide interesting additional results. To achieve this, we will construct a distribution of differences in proportions, by simulation [48,49]. First, the *a priori *distribution is fixed for each of the recovery or success percentages, based on the results available in relation to the treatment in question, the fundamental source for this approach being the studies by Haake *et al*. [23]and by Grotle *et al*. [5]. To define the respective *a priori *distributions (the beta distributions), we shall estimate the frequentist (conventional) confidence intervals associated with these prior data. We shall then choose the (alpha and beta) parameters such that the intervals of maximum density of these distributions will approximately coincide with the confidence intervals obtained by the above-mentioned authors. We shall use the success rates observed in the present study, in the follow up at three months after the start, for each of the experimental and control groups (for each of the comparisons) in order to update the *a priori *information, using Bayesian theory. Ten thousand simulations will be performed to obtain the *a posteriori *empirical distribution of the difference in proportions of success (experimental minus conventional). When we have this distribution, it will be possible to make a non-parametric estimation of the probability of the experimental procedure surpassing the conventional one by at least 10%, and to obtain a suitable confidence interval. Data analysis will be performed using the SPSS v10.5 software package, while the statistical program Epidat 3.0 will be used for the Bayesian analysis.

We intend to carry out a cost-effectiveness analysis; to achieve this, a decision-making tree will be constructed, based on the analysis of data obtained from this clinical trial. It will be presented with a decision-node box in which the choice of one path or another will be in the hands of the medical professional. A circle will contain the probabilistic nodes, in which situations occur with a given probability and cannot be influenced by the clinic. The terminal nodes represent the end of each of the therapeutic decisions, for which a double evaluation will be made: on the one hand, the effectiveness of the treatment for acute low back pain and, on the other, the direct tangible cost of the therapeutic activities considered.

By comparing the results obtained by means of each approach, we can calculate the cost, the marginal cost, the effectiveness and the marginal effectiveness. Finally, we will calculate the marginal cost-effectiveness parameter, as follows: (Cost of approach B - Cost of approach A)/(Effectiveness of approach B - Effectiveness of approach A).

A sensitivity analysis will be carried out, by which we shall obtain the results for the different outset hypotheses, changing the values of the variables or critical factors that present uncertainty. The different therapeutic approaches can be studied by means of an incremental analysis, consisting of dividing the increased cost of a given approach by the increased effectiveness achieved. All the analyses will be made without revealing the blinding of the different branches of the study.

### Sample size

We decided to use a sample size of n = 70 patients in each group. This is the approximate size produced by accepting a probability of 0.05 of committing a type I error, assuming a power of 80% and a ratio of group A/Group D = 1, with a CRI success rate of 85% for Group A (true acupuncture) and of 63% for Group D (conventional treatment). These percentages were taken from the results of a prior pilot experiment, carried out from October to December 2004 at the Dos Hermanas health centre, after two weeks' treatment (unpublished data). We assume a possible dropout rate of 20%. The overall size of the sample will be n = 336 (84 in each group).

### Current status of the trial

Recruiting of the patients began in February 2006 and will continue until December 2006. Follow up is planned to end in January 2008.

## Discussion

Various excellent studies have analysed the effects of acupuncture on chronic acute low back pain, for example Brinkhaus et al. [50]. Nevertheless, rigorous studies have yet to be made to evaluate the efficacy of acupuncture against acute low back pain. Clinical experience has shown that uncomplicated lumbalgia responds rapidly to acupuncture, which achieves a reduction in pain intensity and duration, and also contributes to the prompt return of patients to their normal working activity.

The standardised treatments used in RCT have been criticised as not reflecting normal clinical practice [51], especially in view of the large variety of styles implemented in the West. Although acupuncture treatment is usually individualised, it depends to a large extent on the pathological process identified by the physician; a good diagnosis is essential in order to identify underlying patterns and to choose the most appropriate treatment. The standard pattern found in cases of non-specific acute low back pain is that known in TCM as *Qi *stagnation and blood stasis (*qi zhi xue yu*), although this may be associated with other fundamental disorders such as Impediment pattern (*bi zheng*) or Kidney *qi *vacuity (*shen qi xu*), among others. The choice of acupuncture points for the study group treated with genuine acupuncture was based on 'distance puncture', taking into consideration the standard categories of acute low back pain according to TCM. The doctors participating in the project were free to select up to 4 points, but were required to justify their decision. Another important consideration is the use of 'dynamic puncture' (*xingzhen*), which in most cases facilitates immediate analgesia.

We chose to include in the study a non-treated group, in order to observe the normal evolution of the problem, the Hawthorn effect and the trend towards the mean. The group that was given placebo acupuncture, completely inert, served to isolate the non-specific effects of acupuncture treatment; this group received the same number of sessions as did the experimental group, the same evaluation procedure, the same physical contact and the impression of having received real acupuncture treatment. The group given false acupuncture (i.e. at non-specific points), on the other hand, served to detect the non-specific effects of the puncture, as any stimulus caused by a needle penetrating the skin may be capable of activating spinal nociceptive neurons and sending an excitatory message to the brain centres, thus causing a non-specific analgesic effect, due to the activation of the pain-suppression system in the spinal cords (diffuse noxious inhibitory controls [26,52-54], among other effects. By definition, a placebo must be inert, and so the method adopted (with no penetration of the skin), we believe, is the most valid one.

## Abbreviations

ABC: Activity based casting

BP: Basic points

CA: Consumption of analgesic

CEAR: Collateral effects and adverse reactions

CR: Consumption healthcare recourses

CRI: Clinical relevant improvement

DNIC: Diffuses noxious inhibitory control

DRB: Data record book

EQ-5D: EuroQol 5D

ETCS: Expectation and treatment credibilitu scale

FABQ: Fear advoidance beliefs questionnaire

GP: General Practitioner

IP: individualised points

IPP: Improvement perceived by patient

ITT: Intention to treat

NSAID: Non-steroid anti-inflammatory drug

PP: Per protocol

RMQ: Roland-Morris questionnaire

SP: Specific points

SSPA: Andalusian Public Health System

TCM: Traditional Chinese Medicine

VAS: Visual analogue scale

VBP: Verification of blinding of the patients

## Competing interests

The author(s) declare that they have no competing interests.

## Authors' contributions

Conception and design: J. Vas. Revision of the different versions of the study protocol: J. Vas, E. Perea-Milla, C. Mendez, L. C. Silva. Substantial contributions to the conception and design of the digital data record: A. Herrera Galante, J. M. Aranda Regules, D. M. Martinez Barquin, I. Aguilar y V. Faus. All authors have read and approved the final manuscript.

The study protocol was developed in 2004 and is funded by the *Ministerio de Salud y Consumo – Instituto de Salud Carlos III *(File No. PI051191).

## Pre-publication history

The pre-publication history for this paper can be accessed here:


